# Conventional and Computational Flow Cytometry Analyses Reveal Sustained Human Intrathymic T Cell Development From Birth Until Puberty

**DOI:** 10.3389/fimmu.2020.01659

**Published:** 2020-08-04

**Authors:** Marieke Lavaert, Brecht Valcke, Bart Vandekerckhove, Georges Leclercq, Kai Ling Liang, Tom Taghon

**Affiliations:** Department of Diagnostic Sciences, Faculty of Medicine and Health Sciences, Ghent University, Ghent, Belgium

**Keywords:** human thymopoiesis, aging, computational flow cytometry, puberty, thymic involution

## Abstract

The thymus is the organ where subsets of mature T cells are generated which subsequently egress to function as central mediators in the immune system. While continuously generating T cells even into adulthood, the thymus does undergo involution during life. This is characterized by an initial rapid decrease in thymic cellularity during early life and by a second age-dependent decline in adulthood. The thymic cellularity of neonates remains low during the first month after birth and the tissue reaches a maximum in cellularity at 6 months of age. In order to study the effect that this first phase of thymic involution has on thymic immune subset frequencies, we performed multi-color flow cytometry on thymic samples collected from birth to 14 years of age. In consideration of the inherent limitations posed by conventional flow cytometry analysis, we established a novel computational analysis pipeline that is adapted from single-cell transcriptome sequencing data analysis. This allowed us to overcome technical effects by batch correction, analyze multiple samples simultaneously, limit computational cost by subsampling, and to rely on KNN-graphs for graph-based clustering. As a result, we successfully identified rare, distinct and gradually developing immune subsets within the human thymus tissues. Although the thymus undergoes early involution from infanthood onwards, our data suggests that this does not affect human T-cell development as we did not observe significant alterations in the proportions of T-lineage developmental intermediates from birth to puberty. Thus, in addition to providing an interesting novel strategy to analyze conventional flow cytometry data for the thymus, our work shows that the early phase of human thymic involution mainly limits the overall T cell output since no obvious changes in thymocyte subsets could be observed.

## Introduction

The thymus is the organ where bone marrow-derived thymic progenitors undergo stepwise developmental changes to generate multiple distinct subsets of mature T cells. Subsequently, these naïve T cells egress from the thymus to peripheral lymphoid organs and function as central mediators of the immune system. Thymic involution is an evolutionary process that is conserved in almost all vertebrates (1). This involves regression of the thymus with disruption of the structural integrity that is important to support T-cell development. As a result, the T cell output decreases and this is reflected by a reduction in thymic cellularity and overall size of the organ. Based on the kinetics of thymic involution, two phases of thymic regression have been proposed (2). The first phase involves a rapid decrease in thymic cellularity during early life. In contrast to that, the second phase begins in adulthood in which thymic involution proceeds age-dependently at a steady state. At this stage, age-related declines in T cell output are contributed by both the progressive involuting thymic stromal compartment and intrinsic developmental defects in myeloid-biased thymic progenitors which originate from the aging bone marrow (3–8). Ultimately, thymic involution restricts the diversity of the peripheral T cell repertoire and leads to deterioration of the immune system in the elderly.

Studies in the mouse system have been crucial to advance our understanding of thymic involution. In this species, T cell development after birth is a continuous process that leads to the build-up of thymocytes until the onset of puberty. Following that, the first phase of murine thymic involution begins at 4–7 weeks of age when thymic cellularity decreases sharply (9–11). Indeed, the rise in sex steroid levels during puberty has an established causative role in thymic involution (12). In contrast to the mouse, studies examining the human thymus revealed that human T cell development is a dynamic process that fluctuates before the puberty period (13, 14). Within 3–4 weeks after birth, the thymic cellularity of neonates remains low. Thereafter, human T cell development raises, and the thymus of infants reaches its maximum cellularity at 6 months of age (14). The early onset of human thymic involution prior to puberty is corroborated by the morphological alterations found in the thymus from 1 year of age (15). Nevertheless, Weerkamp and colleagues found that the cellular composition of the thymus, in consideration of the different subsets of developing thymocytes, remains similar from 6 months to 8 years of age. Hence, involution in the thymus of infants does not impede its ability to sustain T-cell development in early childhood. Sex steroid ablation has been shown to improve human thymic and hence immune regeneration (16, 17). However, the physiological changes of human thymic composition from birth until the onset of puberty, which is around the age of 11.5 years old, remains unclear (18). To address this, we performed multi-color flow cytometry on thymic samples collected from birth to 14 years of age in order to examine changes in population distribution at critical developmental stages of human T cell development. In addition, we also studied age-associated changes in mature T and non-T lineage populations that reside in the thymus. In acknowledgment of the inherent limitations posed by conventional flow cytometry analysis, we implemented a novel computational analysis pipeline that is adapted from single-cell transcriptome sequencing data analysis to present our data.

## Results

### The Human Postnatal Thymus Is Characterized by An Increased Frequency in B Cells Following the Onset of Thymic Involution

**Figure 1:**
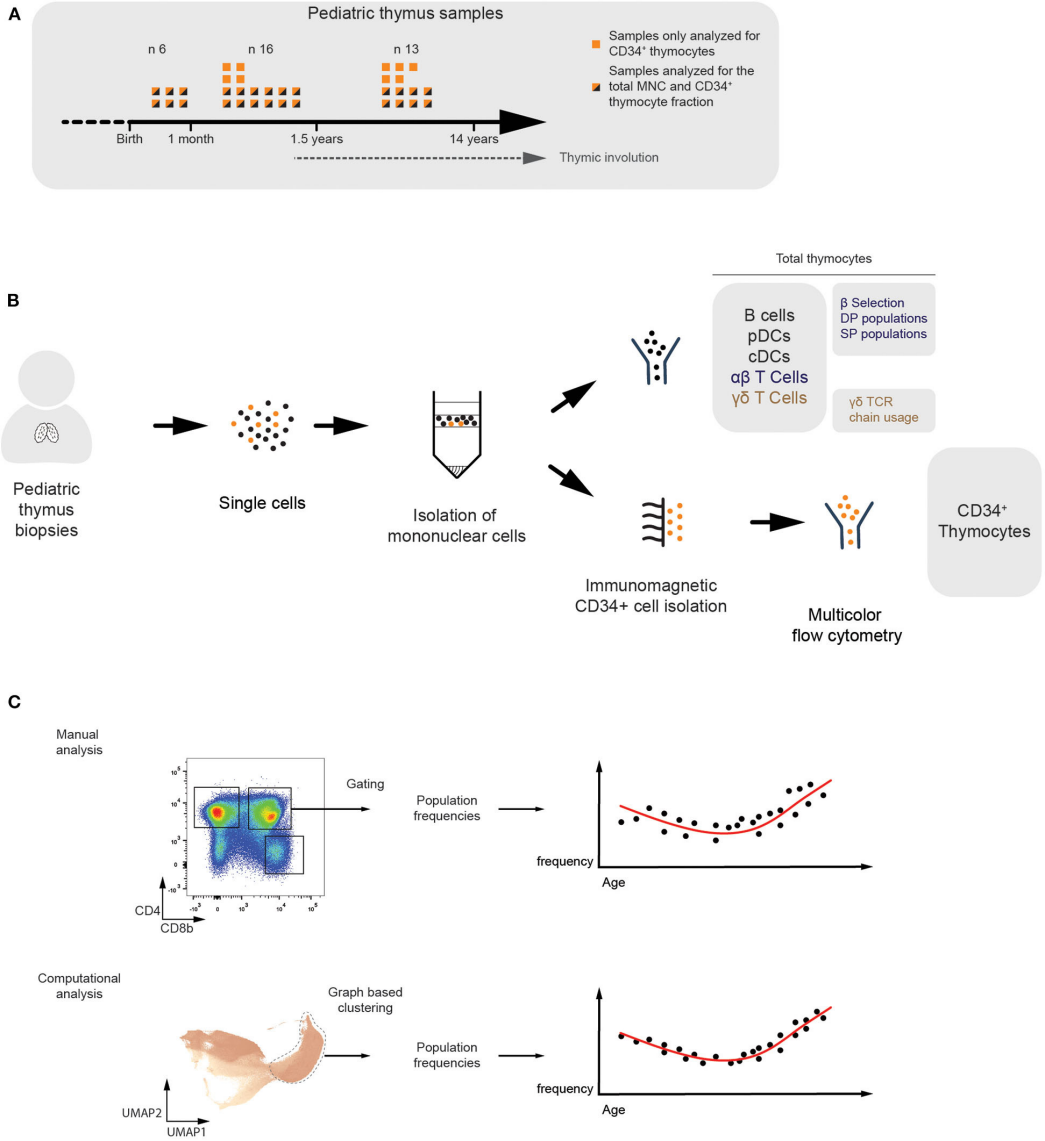
Multicolor flow cytometric analysis of human thymocyte populations of different ages. **(A)** Overview of the thymus samples ranging from birth to puberty. Flow cytometry analyses were performed on the total mononuclear cell fraction of 26 different donors (*n* = 26). From these, and from an additional nine different donors, separate CD34^+^-enriched fractions were analyzed in more detail (*n* = 35). **(B)** Schematic overview visualizing how the total MNC (*n* = 26) and CD34^+^ (*n* = 35) fractions were isolated from the human pediatric thymus samples and analyzed using multicolor flow cytometry. The immune subsets being studied were specified. **(C)** Overview of both the manual and computational analysis strategies in which populations are identified by either manually defined gates (top) or graph-based clusters (bottom) in order to allow observation of age-dependent changes in the population frequency.

To study the age-dependent fluctuations in the frequencies of thymic immune cells, we collected 35 human thymus samples ranging from birth to 14 years of age. The CD34^+^ thymocyte fraction was isolated from all the samples and the total mononuclear cell fraction (MNC) was studied from 26 of them (Figure 1A). Multicolor flow cytometry was applied to identify different subsets of thymic progenitors present in the CD34^+^-enriched fractions. For the MNC fractions, we immunophenotyped developing and mature T-lineage subsets, spanning both the αβ and γδ branches, and mature non T-lineage cells (Figure 1B). The flow cytometry data generated from these samples was analyzed using manually defined gates. In addition, we applied a computational approach, based on established single cell RNAseq pipelines (19), which consists of two steps. First, in order to improve computational throughput, compensated and biexponentially transformed data is subsampled and pooled, either in a random manner or by using geometric sketching (20) which allows for enrichment of rare subpopulations, or these subsampling methods can be combined as was done in all the following analyses. Subsequently, a k-nearest neighbor (KNN) graph was constructed and Leiden clustering was performed (21) to allow identification of populations within the data, which in turn were visualized using an UMAP manifold (22) (Figure 1C).

**Figure 2:**
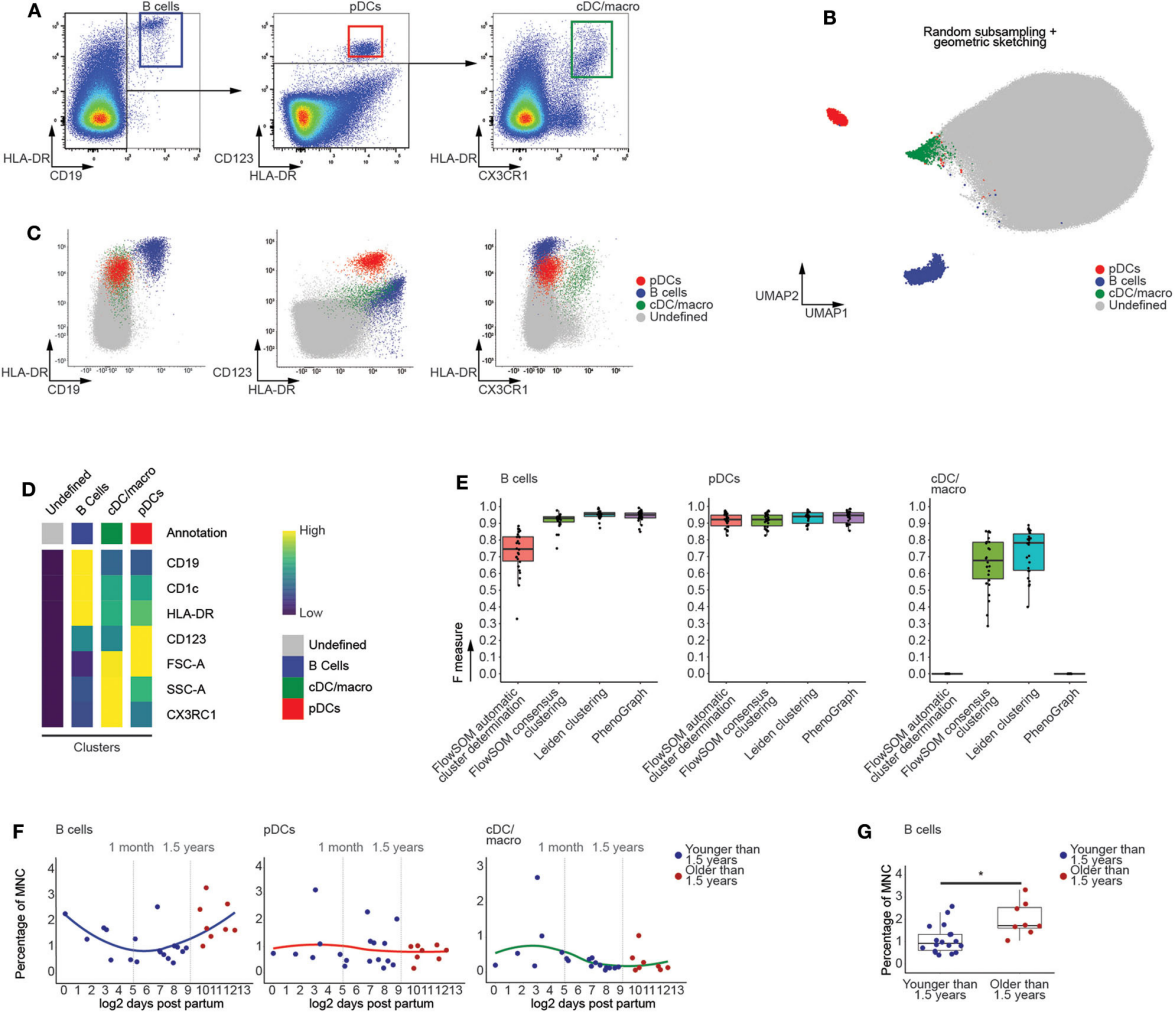
Computational flow cytometry revealed an increased frequency of B cells following the onset of human thymic involution. **(A)** Manual identification of immune subsets within the CD45^+^ thymocyte population, representative for 26 samples. **(B)** Annotated UMAP visualization of thymocytes derived from 26 samples following subsampling. **(C)** Scatterplots of biexponentially transformed data visualizing the populations identified in **(B)** using Leiden clustering. **(D)** Heatmap visualizing scaled median fluorescence indices (MFI) of the annotated clusters. **(E)** Boxplots displaying the F measures for each of the MNC thymocyte samples (*n* = 26) per algorithm and population. **(F)** Dot plots visualizing age-dependent changes within thymocytes populations, with samples colored in blue (<1.5 years) or red (>1.5 years). A loess curve (span = 1) was fitted through the data in order to visualize the trend. **(G)** Boxplots visualizing discrete changes in the two age categories defined in **(F)**. * indicates *q* values < 0.05 based on linear regression between discrete groups.

Using manually defined gates, we examined the CD45^+^ fraction of human thymic MNC in order to identify populations of CD19^+^HLA-DR^+^ B cells, HLA-DR^+^CD123^+^ plasmacytoid dendritic cells (pDCs) and HLA-DR^+^CX3CR1^+^ conventional dendritic cell and macrophages (cDC/macro) (Figure 2A) and HLA-DR^+^CD14^+^ monocytes (Figure S1A) (23, 24). These populations were used to assess the performance of our graph-based clustering approach, which appeared to successfully identify the distinct populations (Figure 2B and Figure S1B) and which recapitulated the phenotype of manually identified mature non T-lineage subsets based on both the biexponentially transformed scatterplots (Figure 2C and Figure S1C) and the median fluorescent indices (MFIs) of each of the annotated clusters (Figure 2D). Further assessment of our methods performance was done by calculating F measures for each population, taking both the precision and recall of the manually defined populations into account. This also allowed comparisons with well-established state-of-the-art methods such as PhenoGraph and FlowSOM (25, 26), of which the latter can both automatically detect an optimal number of clusters or return a specific number of clusters (Figure 2E and Figure S1D). This comparison revealed that our approach either is equally or even more performant than FlowSOM or PhenoGraph. In addition, the frequencies of these manually defined populations were not affected by the initial subsampling of the data (Figure S1E), which was also the case for the populations that we cover later (Figure S2). This suggests that our computational approach was not only able to identify relevant immune subsets, but also allows assessment of age-dependent changes in population frequencies.

As the onset of thymic involution occurs after 1 year of age (15), we categorized the samples in two groups, being younger or older than 1.5 years of age, and used linear regression to test for changes in population frequency. This approach revealed that the population of computationally identified B cells significantly increases in frequency after 1.5 years of age (Figures 2F,G), consistent with earlier reports on increased B cell numbers in the aging thymus (27) which validates our choice to generate two groups based on this age. In contrast, the myeloid populations appeared largely unaffected by aging prior to puberty (Figures 2F,G and Figure S3), in agreement with recently published findings (28). Together, these findings not only validate the quality of our data, but also demonstrate the capability of our approach to robustly identify rare populations, with frequencies as low as 0.2%, within the generated flow cytometric data.

### Developing CD34^+^ Human Thymocytes Frequencies Are Unaffected by Physiological Growth Leading To Puberty

**Figure 3:**
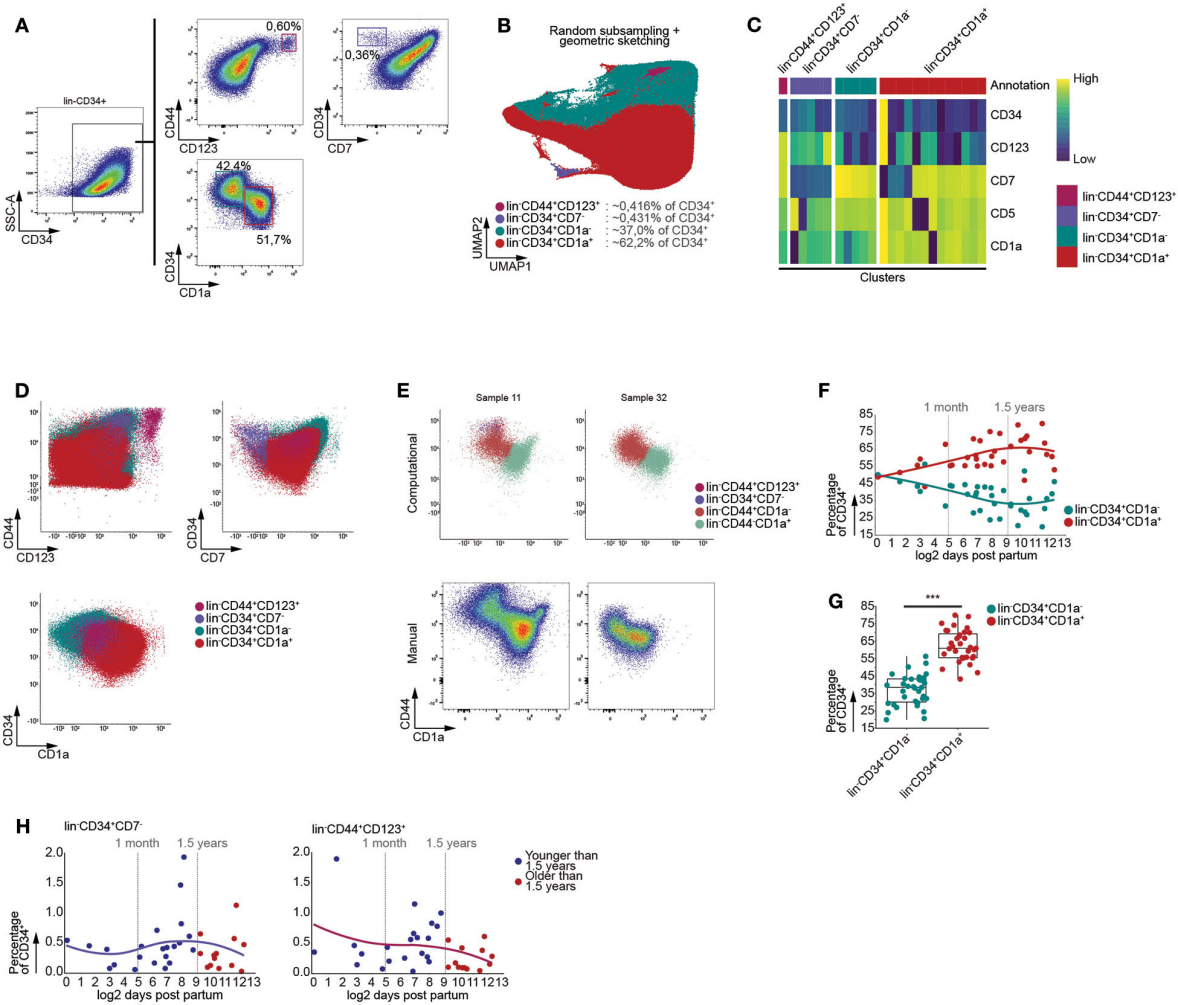
Graph based clustering allows identification of gradual developmental changes within CD34^+^ thymocytes. **(A)** Schematic overview of immune subset identification using manual gating, representative for 35 samples. Scatterplots visualize biexponentially transformed data. **(B)** Annotated UMAP visualization of subsampled, both randomly and by geometric sketching, CD34 enriched thymocytes, derived from 35 samples. **(C)** Heatmap visualizing the scaled MFIs of each cluster grouped according to the annotated population. **(D)** Scatterplots of biexponentially transformed data visualizing the annotated populations allowing comparison between the manual gating **(A)** and computationally defined populations. **(E)** Visualization of biexponentially transformed data displaying computationally defined populations based on CD44 expression (Top), highlighting difficulties with defining manual gates (Bottom). **(F)** Dot plots visualizing population frequencies with samples ordered according to log2 transformed age. Observations are colored according to subset corresponding to either the lin^−^CD34^+^CD1a^−^ or lin^−^CD34^+^CD1a^+^. A loess curve (span = 1) was fitted through the data in order to visualize the trend. **(G)** Boxplots visualizing changes in frequency of MNC between the lin^−^CD34^+^CD1a^−^ or lin^−^CD34^+^CD1a^+^. Statistical significance denoted by *** indicates q values < 0.0001 based on a paired *t*-test between discrete groups. **(H)** Dot plots visualizing the frequency of MNC with samples ordered according to the log2 of days after birth. Samples derived from patients younger than 1.5 years of age colored in blue and those older than 1.5 years of age in red. A loess curve (span = 1) was fitted through the data in order to visualize the trend.

During human life, the thymus is continuously seeded by bone marrow derived multipotent progenitor cells. These CD34^+^ precursors lack high expression of CD7 and can give rise to T cells and to a lesser extent pDCs (29–31). In order to assess age-dependent effects on these initial stages of T cell development, we enriched the CD34^+^ thymocytes and performed multi-color flow cytometry. Using manual gating, we were able to identify an immature CD3^−^CD4^−^CD19^−^CD56^−^(lin^−^)CD7^−^CD34^+^ progenitor population, both the uncommitted lin^−^CD34^+^CD1a^−^ and T-lineage committed lin^−^CD34^+^CD1a^+^ thymocyte populations, and lin^−^CD44^+^CD123^+^ DC progenitors (Figure 3A) (30, 32, 33).

We were able to recover these populations from the lin^−^CD34^+^ thymocytes fraction following exclusion of CD44 from the data, as it follows a similar expression pattern to CD34 and has been inversely associated with T-lineage commitment (34), and performing batch correction with linear regression, prior to generating a KNN-graph and clustering (Figures 3B–D). This indicated that our approach was capable of identifying highly distinct and rare subpopulations, as well as more subtle changes within relatively homogenous populations. Calculating F measures for each population further confirmed our approach to be capable of faithfully recovering these populations and to be equally as performant or outperform well established algorithms (Figure S4A), such as FlowSOM and PhenoGraph.

Due to the nature of our computational approach, which allowed for both the simultaneous analysis of multiple samples and batch correction using linear regression, we were able to consistently identify particular populations despite inter-sample variation. In case of our data, incorporation of CD44 while excluding CD34 prior to KNN-graph generation allowed us to robustly isolate the lin^−^CD44^+^CD1a^−^ and lin^−^CD44^−^CD1a^+^ populations (Figure 3E), regardless of clear differences between samples with respect to the expression level of specific cell surface markers.

As an increase in B cell frequency could be observed from 1.5 years of age onwards, following the onset of thymic involution, we assessed whether or not this was the case for the subpopulations we identified within the CD34^+^ fraction of human thymocytes. However, no significant age-dependent changes within the lin^−^CD34^+^CD1a^−^, lin^−^CD34^+^CD1a^+^ (Figure S4B), lin^−^CD44^+^CD1a^−^ and lin^−^CD44^−^CD1a^+^ (Figure S4C) thymocyte populations could be observed using linear regression. These findings were confirmed as we were able to observe significant differences in the proportions of lin^−^CD34^+^CD1a^−^ and lin^−^CD34^+^CD1a^+^ (Figures 3F,G), and lin^−^CD44^+^CD1a^−^ and lin^−^CD44^−^CD1a^+^ thymocytes (Figures S4D,E), regardless of age. Similarly, the lin^−^CD44^+^CD7^−^ and lin^−^CD44^+^CD123^+^ populations displayed no significant age-dependent differences despite the onset of thymic involution (Figure 3H). Using these data, we were able to validate that our computational approach is capable of identifying both highly distinct and gradually transitioning populations in a manner that disregards inter-sample variation, enabling the identification of age-dependent effects or lack thereof following the onset of thymic involution.

### Thymic Involution Does Not Affect Major Developmental Intermediates During Thymopoiesis

**Figure 4:**
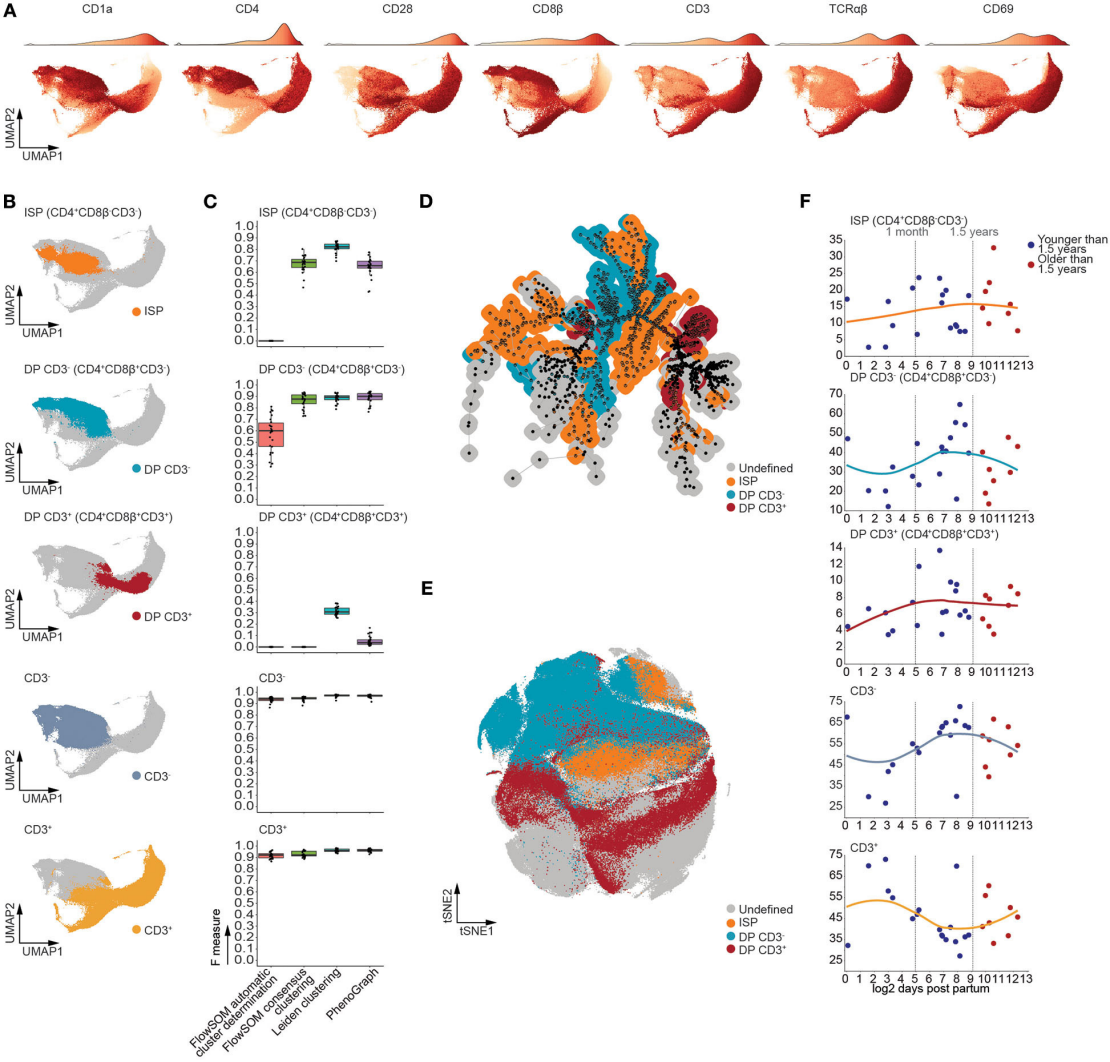
T cell developmental intermediates were consistently detected from birth to puberty in the human thymus. **(A)** UMAP visualizing expression patterns of distinct markers. The accompanying color bar is visualized as the density of the number of cells on top of each UMAP. **(B)** UMAP visualizing distinct populations of developmental intermediates detected using Leiden clustering. **(C)** Boxplots of the F measures calculated for each of the MNC samples (*n* = 26) per analysis method and population. **(D)** Annotated MST visualizing distinct populations of developmental intermediates as detected in **(B)**. **(E)** Annotated tSNE visualizing distinct populations of developmental intermediates as detected in **(B)**. **(F)** Dot plots visualizing the frequency of MNC with samples ordered according to the log2 of days after birth. Samples derived from patients younger than 1.5 years of age colored in blue and those older than 1.5 years of age in red. A loess curve (span = 1) was fitted through the data in order to visualize the trend.

As our computational approach allows for robust identification of developmental intermediates, we generated a UMAP manifold visualizing all major stages of TCRαβ T-cell development (Figure 4A), with the exception of CD34^+^ progenitors, which constitute ~1% of the human postnatal thymus. Using multiple rounds of Leiden clustering, we isolated the CD4^+^CD8β^−^CD3^−^ immature single positive (ISP), the CD4^+^CD8β^+^CD3^−^ and CD4^+^CD8β^+^CD3^+^ double positive, and the total CD3^−^ and CD3^+^ populations (Figure 4B and Figure S5A). Despite the complexity of this developmental trajectory, this approach was able to faithfully recapitulate these populations (Figure S5B). One exception to this was the double positive CD3^−^ (CD4^+^CD8β^+^CD3^−^) population (Figure S5C) which contained a minor fraction of CD4^−^CD8β^+^CD3^−^ cells, randomly varying between 0.25 and 2.5% of MNC (Figure S5D) and which could be observed by both manual or computational analysis (Figure S5E). Nevertheless, for the main subsets, these findings were further confirmed by calculating F measures, which not only validated our approach but also revealed our method to be equally or more performant compared to FlowSOM and PhenoGraph (Figure 4C), in line with our previous observations. Moreover, visual inspection of the t-distributed Stochastic Neighbor Embedding (tSNE) and minimal spanning tree (MST), used to visualize PhenoGraph and FlowSOM, respectively, revealed the developmental intermediates to be scattered (Figures 4D,E) rather than being closely grouped as in our UMAP visualization (Figure 4B) (35). In line with our findings that the onset of thymic involution does not affect the frequencies the CD34^+^ thymocyte subpopulations, no significant age-dependent changes in population frequencies could be observed following 1.5 years of age using linear regression in Figure 4F, suggesting that the onset of thymic involution does not initially affect the frequency of major developmental intermediates during human TCRαβ T cell development.

### The Human Thymus Maintains Generation of Mature αβ and γδ T Cells From Birth To Puberty

**Figure 5:**
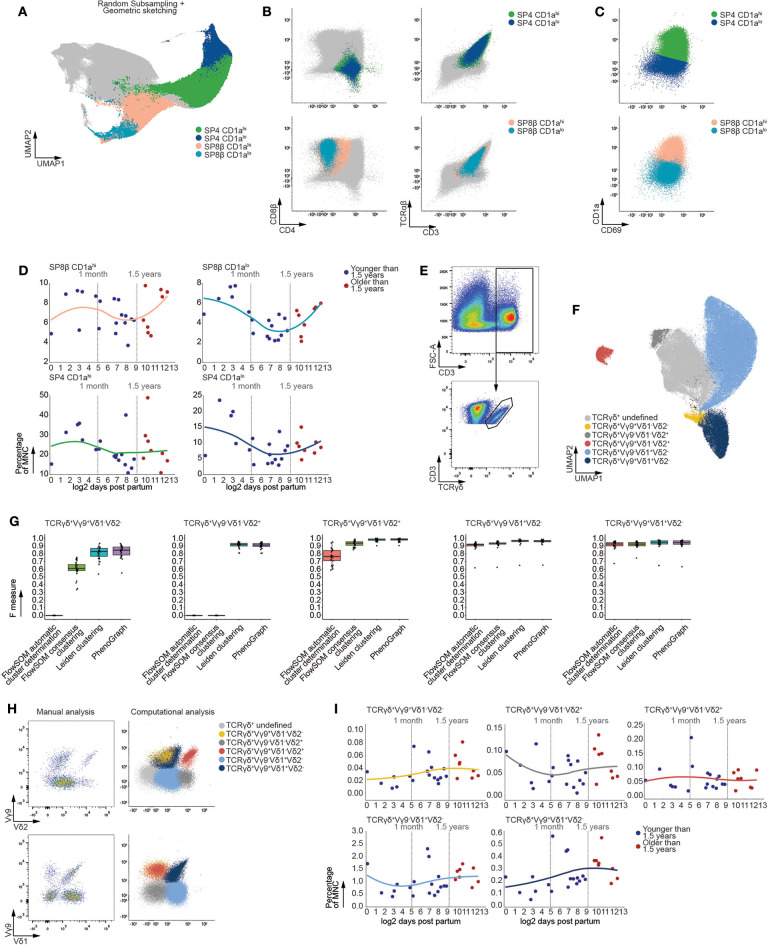
The frequencies of mature T cells populations remain stable following thymic involution. **(A)** Fully annotated UMAP visualizing subpopulations of mature SP CD4^+^ and SP CD8β^+^ T cells detected using Leiden clustering. **(B)** Scatterplots of biexponentially transformed data visualizing the populations detected in **(A)** using relevant markers. **(C)** Scatterplots of biexponentially transformed data visualizing subpopulations of either SP CD8β^+^ or SP CD4^+^ T cells based on decreased expression of CD1a. **(D)** Dot plots visualizing the frequency of MNC with samples ordered according to the log2 of days after birth. Samples derived from patients younger than 1.5 years of age colored in blue and those older than 1.5 years of age in red. A loess curve (span = 1) was fitted through the data in order to visualize the trend. **(E)** Scatterplots of biexponentially transformed data visualizing manual gating used to isolate CD3^+^TCRγδ^+^ T cells, representative for 26 samples. **(F)** Fully annotated UMAP visualizing subpopulations of mature CD3^+^TCRγδ^+^ T cells (*n* = 26) based on variable expression of Vδ1, Vδ2, and Vγ9 TCR chain expression detected using Leiden clustering. **(G)** Boxplots visualizing the F measures for each of the MNC samples (*n* = 26) per analysis method and population. **(H)** Scatterplots visualizing biexponentially transformed data covering the CD3^+^TCRγδ^+^ subpopulations obtained from a single sample representative for 26 samples (left) and CD3^+^TCRγδ^+^ subpopulations obtained from the simultaneous analysis of 26 samples (right). **(I)** Dot plots visualizing the frequency of MNC with samples ordered according to the log2 of days after birth. Samples derived from patients younger than 1.5 years of age colored in blue and those older than 1.5 years of age in red. A loess curve (span = 1) was fitted through the data in order to visualize the trend.

Following expression of a functional αβ TCR, developing CD3^+^ thymocytes bifurcate toward either the single positive (SP) CD4^+^ or SP CD8β^+^ branch (Figure 5A). Maturation of these SP populations, from an immature naïve to a mature naive phenotype, is marked by a decrease in CD1a expression prior to thymic egression (36, 37). Using Leiden clustering, we distinguished these SP populations (Figure 5B) and also identified developmental intermediates within the SP CD8β^+^ thymocytes, being the SP CD8β^+^CD1a^hi^ and SP CD8β^+^CD1a^lo^ subsets (Figure 5C). In order to circumvent differences in clustering between both branches of αβ T cells, logistic regression was used to infer these mature SP CD8β^+^ populations onto the SP CD4^+^ branch, allowing accurate identification of the SP CD4^+^CD1a^hi^ and SP CD4^+^CD1a^lo^ developmental intermediates (Figure 5C and Figure S6A). These populations were then validated using F measures, revealing high correspondence with their manually defined counterparts, and allowing us to confirm our approach to be equally or more performant than FlowSOM and PhenoGraph (Figure S6B). Moreover, in line with our earlier analysis of the major T-lineage developmental stages, both the MST and tSNE visualizations used for FlowSOM or PhenoGraph were not capable of tightly arranging these populations in contrast to the UMAP manifold used here (Figures S6C,D) (35).

In agreement with the developmental intermediates precluding bifurcation of the αβ T cells, linear regression revealed no significant changes in population frequency following the onset of thymic involution (Figure 5D). In addition, examination of the total SP population frequencies did not reveal significant differences prior to and after 1.5 years of age (Figure S6E).

Prior to the expression of a functional αβ TCR, a minor fraction of thymocytes branch away from the αβ T-lineage to give rise to γδ T cells, which constitute approximately 2% of all thymocytes regardless of age (Figure S6F). This minor population of T cells is characterized by limited variation in their TCR chain usage compared to αβ T cells. Consequently, we stained MNC with antibodies directed at the Vδ1, Vδ2, and Vγ9 TCR chains and assessed age-dependent effects in these corresponding γδ T cell subpopulations. As this panel was optimized to assess variation within these rare γδ T cells, examination of the entire MNC fraction was not possible, necessitating isolation of CD3^+^TCRγδ^+^ cells using manual gating (Figure 5E). Leiden clustering robustly identified populations of TCRγδ^+^Vγ9^+^Vδ1^+^Vδ2^−^, TCRγδ^+^Vγ9^+^Vδ1^−^Vδ2^+^, TCRγδ^+^Vγ9^−^Vδ1^+^Vδ2^−^, TCRγδ^+^Vγ9^+^Vδ1-Vδ2^−^ and TCRγδ^+^Vγ9^−^Vδ1^−^Vδ2^+^ thymocytes within the CD3^+^TCRγδ^+^ cells (Figure 5F), which corresponded well to the manually detected populations and proved our approach to be equally or more performant compared to current algorithms (Figure 5G). Moreover, our approach allowed for enrichment of rare populations as clustering was performed on all 26 samples simultaneously (Figure 5H), which is often hampered during manual gating. Despite the robust detection of these γδ T cell subpopulations, no significant age-dependent effects could be observed within each subpopulation following the onset of thymic involution (Figure 5I). This lack of age-dependent effects within populations that constitute both the mature αβ and γδ T cell further confirmed the lack of observed variation in frequency within the developing T-lineage cells.

## Discussion

The lack in improved therapies that are directed at the recovery of T cell development following hematopoietic stem and progenitor cell transfer in the case of immunodeficiencies or malignancies can, in part, be attributed to the limited understanding of how aging affects this biological process. In order to study these age-dependent effects, we collected the MNC fraction from 26 thymic biopsies and the CD34^+^ fraction from an additional nine thymic biopsies, ranging from birth to puberty, obtained from patients undergoing cardiac surgery. Using multi-color flow cytometry, we assessed the frequencies of T-lineage developmental intermediates within the CD34^+^ fraction up until the SP CD4^+^ and SP CD8β^+^ cells, as well as the frequencies of γδ T cells and thymic non T-lineage subsets, which fulfill a supportive role in maintaining thymic homeostasis. Analysis of these data was done computationally by subsampling the data prior to performing consecutive rounds of Leiden clustering, thereby allowing simultaneous isolation of these populations in all samples, while circumventing batch effects. We were able to validate the performance of our computational pipeline using these flow cytometric datasets. In turn, this allowed assessment of age-dependent effects on immune subsets within the human thymus, revealing the human thymus to consistently generate both αβ and γδ T cells and their developmental intermediates despite thymic involution. Moreover, cell types involved in maintenance of thymic homeostasis did not display significant variation, except for B cells which increase in frequency as previously reported (27).

The increasing complexity of data obtained by biologists has necessitated the development of advanced analysis methods. In the field of flow cytometry, this has resulted in the generation of algorithms such as flowSOM (26), which implements self-organizing maps to reduce the number of observations and effectively decrease computational costs, and PhenoGraph (25), which approximates the data using KNN-graphs and subsequently clusters using the Louvain algorithm allowing the analysis of thousands of individual cells. Recently, single cell RNAseq has become increasingly popular, spurring the development of even more efficient clustering methods such as the Leiden clustering (21), dimensionality reduction techniques such as UMAP (22) and subsampling methods that preserve the topology of the original data, such as geometric sketching (20). Here, we combined these methods to perform an initial subsampling of the flow cytometric data, effectively reducing the computational cost, followed by the generation of a KNN-graph and Leiden clustering. The initial subsampling was validated using manually defined subpopulations, demonstrating that neither random subsampling nor geometric sketching negatively influences the approximation of population-based frequencies, confirming the validity of this initial step which is essential to reduce computational costs. Future implementation of these methods will depend on the research question at hand as random subsampling does not alter population frequencies, thereby resulting in a slightly more reliable approximation of population frequencies while being limited in the recovery of rare populations. This contrasts with geometric sketching that enriches for rare populations. By implementing both methods for validating Leiden clustering, we were able to recover highly distinct, yet rare subpopulations of non T-lineage populations that recapitulated the manually identified subsets. In addition, Leiden clustering was capable of isolating developmental intermediates within the CD34^+^ thymocyte fraction and the MNC fraction, as well as subsets of mature αβ and γδ T cells in the MNC fraction. This graph-based clustering approach appeared to be advantageous to overcome biological differences, demonstrating robust identification of for instance lin^−^CD44^+^CD1a^−^ and lin^−^CD44^−^CD1a^+^ populations despite clear inter-sample variation. Moreover, batch effects, attributable to technical variations, could be overcome by implementing linear regression, effectively enabling simultaneous analysis of all CD34^+^ samples. In conclusion, our analysis pipeline can overcome technical effects by enabling batch correction, allowing the simultaneous analysis of multiple samples, while limiting the computational cost by subsampling and relying on KNN-graphs that open the possibility of graph-based clustering which enables the robust identification of both distinct and gradually developing immune subsets.

The human thymus has been reported to undergo involution in two phases, with the first one characterized by a decrease in cellularity from 6 months onwards and already undergoing histological changes following the first year of childhood. Therefore, we assessed age-dependent variation within populations frequencies of non T-lineage cells which revealed an increase in the B cell frequency following the onset of thymic involution and which was previously reported to be involved in thymic immune surveillance during aging (27). These findings did not only validate our data quality, despite the modest statistical power, but also revealed a biologically relevant timepoint within our samples age range which coincided with the previously reported histological changes. Rather than describing potential fluctuations within our data, as previously done (14), we assessed whether the onset of thymic involution influences thymocyte population frequencies by defining discrete groups based on the increase in B cell frequency. This effectively disregarded fluctuations in population frequencies attributable to childbirth within the first month of life. In contrast to B cells, myeloid population frequencies remained stable regardless of age, in line with earlier findings based on scRNAseq data (28) and consistent with their role in antigen presentation and maintenance of thymic homeostasis. Indeed, detailed analysis of the immature thymocyte populations within the CD34^+^ fraction did not reveal age-dependent fluctuations in their corresponding frequencies. In contrast, both the lin^−^CD34^+^CD1a^−^ and lin^−^CD34^+^CD1a^+^, and the lin^−^CD44^+^CD1a^−^ and lin^−^CD44^−^CD1a^+^ populations were found to be significantly different from each other regardless of their age, suggesting that aging does not affect the distribution of committed CD34^+^ progenitors. In addition, assessment of the MNC fraction did not reveal significant changes in population frequencies of T cell developmental intermediates, despite the fact that the human thymus is characterized by changes in structural integrity from the age of 1 year and onwards. Furthermore, no age-dependent changes could be observed within the mature αβ and γδ T cells subpopulations. Together, even though the human thymus undergoes structural changes during childhood and is characterized by a decrease in cellularity, our findings suggest that this does not result in a disruption or significant disturbance in the production of T cells, nor in changes in the kinetics or survival/proliferation of specific developmental intermediates. This may therefore imply that therapeutic efforts, directed at the recovery of T cells in case of immunocompromised children, should not only be focused on the T cells themselves, but also on their microenvironment which possibly limits their numbers due to niche availability.

## Methods

### Isolation of MNC and CD34^+^ Progenitors From Postnatal Thymus

Thymus from patients undergoing cardiac surgery were obtained and used according to and with the approval of the Medical Ethical Commission of Ghent University Hospital (Belgium). The thymus tissue was mechanically disrupted to obtain a single cell suspension. After overnight incubation at 4°C, MNC were derived from the cell suspension using a Lymphoprep density gradient and cryopreserved. For flow cytometry analysis of CD34^+^ thymocytes, frozen MNC fractions derived from all samples were thawed for CD34 enrichment using magnetic activated cell sorting (Miltenyi Biotec) according to the instructions of the manufacturer prior to staining. For flow cytometry analysis of developmental intermediates of T-lineage, mature T and non-T lineage populations, frozen MNC fractions from 26 samples were thawed for direct staining.

These samples were stained for flow cytometric analysis using the following antibodies: anti-CD34 PerCP-eFluor710 (Cat# 46-0349-42, RRID:AB_2016673), anti-CD4 FITC (Cat# 11-0042-82, RRID:AB_464896), anti-CD7 Alexafluor700 (Cat# 561603, RRID:AB_10898348), anti-CD3-APC (Cat# 300411, RRID:AB_314065), anti-CD14-APC (Cat# 130-110-576, RRID:AB_2655048), anti-CD19-APC (Cat# 17-0193-82, RRID:AB_1659676), anti-CD56-APC (Cat# 341025, RRID:AB_400558), anti-CD123 PE-Cy7 (Cat# 306009, RRID:AB_493577), anti-CD44 PE (Cat# 12-0441-82, RRID:AB_465664), anti-CD5 AmCyan (Cat# 555350, RRID:AB_395754), anti-CD1a Pacific Blue (Cat# 48-0019-42, RRID:AB_1907358), anti-CD45 PerCP-Cy5.5 (Cat# 304027, RRID:AB_1236444), anti-CD19 FITC (Cat# 302205, RRID:AB_314235), anti-HLA-DR APC-Cy7 (Cat# 327017, RRID:AB_2566388), anti-CD1c AlexaFluor700 (Cat# 331529, RRID:AB_2563656), anti-CLEC9A APC (Cat# 353805, RRID:AB_2565518), anti-CX3CR1 PE (Cat# 355703, RRID:AB_2561680), anti-CD14 FITC (Cat# 367115, RRID:AB_2571928), anti-CD16 PE (Cat# 561313, RRID:AB_10643606), anti-CD11c Pacific Blue (MHCD11C28, RRID:AB_10375450), Anti-CD28 PerCP-Cy5.5 (Cat# 302921, RRID:AB_2073719), anti-CD69 FITC (Cat# 310903, RRID:AB_314838), anti-CD3 APC-Cy7 (Cat# 300425, RRID:AB_830754), anti-CD27 AlexaFluor700 (Cat# 356415, RRID:AB_2562515), anti-TCRαβ APC (Cat# 306717, RRID:AB_10612747), anti-CD25 PE-Cy7 (Cat# 25-0259-42, RRID:AB_1257140), anti-CD8β PE (Cat# 641057, RRID:AB_1645747), anti-CD4 Amcyan (Cat# 300501, RRID:AB_314069), anti-CD1a Pacific Blue (Cat# 48-0019-42, RRID:AB_1907358), anti-CD34 PerCP (Cat# 343519, RRID:AB_1937270), anti-Vδ1 APC (Cat# 130-119-145, RRID:AB_2733450), anti-Vδ2 PE-Cy7 (Cat# 130-111-129, RRID:AB_2653979), anti-TCRγδ PE (Cat# 130-109-357, RRID:AB_2654033), anti-Vγ9 Pacific Blue (Cat# 130-107-486, RRID:AB_2653945).

### Data Preprocessing

Flow cytometric data was exported and loaded into FlowJo, where the data underwent compensation and biexponential transformation. Using manually defined gates, doublets (FSC-A vs. FSC-W and SSC-A vs. SSC-W), dead cells and debris (FSC-A vs. SSC-A) were excluded from the analysis. For the immunophenotyping of CD34^+^ thymocytes, CD3^+^CD14^+^CD19^+^CD56^+^ lineage cells were also excluded from the analysis. In the case of monocyte and macrophage staining, CD11c^+^CD123^+^ lineage cells were excluded. In addition, all the non T-lineage mature cells were gated for CD45 expression.

From the flowjo workspaces, these preprocessing gates were isolated using the flowWorkspace library in R. Subsequently, each exported fcs file was loaded in R using the read FCS function from the FlowCore library and unwanted cells, based on either gating or because these cells were out the detection limit, were removed. Next, using functions from the FlowCore library, samples were compensated using the compensation function and biexponentially transformed using the logicleTransform function which uses fixed parameters in contrast to the biexponential transformation in FlowJo. For each antibody panel all samples were combined in a Single Cell Experiment object. Finally, z-scores were calculated from these transformed data, allowing the usage of FSC-A and SSC-A during subsampling and KNN-graph generation. For these subsequent steps, specific markers were selected from each panel based on their performance. From the panel used to stain CD34^+^ thymocytes, the CD34, CD44, CD7, CD5, and CD123 markers were retained; from the monocyte/macrophage panel, the CD14, CD16, and HLA-DR markers, in addition to FSC-A and SSC-A parameters were retained; from the B cell and DC panel, the CX3RC1, HLA-DR, CD19, CD1c, and CD123 markers, in addition to the FSC-A and SSC-A parameters were retained; from the γδ T cell panel, the TCRγδ, Vγ9, Vδ1, and Vδ2 markers, in addition to the FSC-A and SSC-A parameters were retained; and from the αβ T cell panel, the CD4, CD8β, TCRαβ, CD3, CD69, CD1a, and CD28 markers were retained.

In case batch correction was performed, the lmFit and residuals.MArrayLM functions from the limma package (38) were used. In order to improve computational efficiency, each sample needed to be subsampled. This was done in both a randomized manner using the sample function and by running the geometric sketching function (20) directly on the transformed data in R using the reticulate package, which enables usage of python modules in R.

For visualization purposes, the data was transformed using the scale_x_flowJo_biexp and scale_y_flowJo_biexp functions from the ggcyto library to allow comparisons between populations derived from manual analysis and computational analysis.

### Data Analysis

These subsampled data were used to generate a UMAP (22) using the uwot library in R. This was done by feeding the z-scores directly in the umap function which ran with the ret_nn and ret_model variables enabled and with “fgraph” specified for the ret_extra variable. The resulting UMAP was used to visualize the data while the connectivities matrix was used for clustering. In order to perform Leiden clustering, the connectivities were used to generate a directed igraph object using reticulate and subsequently used in the find_partition function from the leidenalg module while specifying the RBConfigurationVertexPartition function as partition_type in R using reticulate.

### Benchmarking

The performance of our method was benchmarked using well established methods, such as FlowSOM and PhenoGraph. The FlowSOM pipeline was run using a symmetric 1225 node self-organizing map (SOM). Metaclusters were detected using the MetaClustering function, specifying the method variable as metaClustering_consensus, allowing automatic cluster detection, and the metaClustering_consensus function. For both clustering approaches a maximum of 50 clusters was requested. In order to visualize the FlowSOM results each node was given equal size using the UpdateNodeSize function and plotted using the PlotPies function.

PhenoGraph was run using standard settings. Data visualization was achieved by generating a tSNE without preprocessing the data by principal component analysis.

The performance each method was compared by calculating the F measure for each sample using the manual gating as a reference. This was done using the ConfusionMatrix function, specifying the mode as “prec_recall,” from the caret package.

### Calculation of Frequencies

From the data subsampled using geometric sketching, each sample was subdivided in 10 equal bins. Using the MiniBatchKmeans function from the sklearn module, these bins were then clustered into groups of approximately 500 cells. Using these clusters as a reference, the sampling bias resulting from geometric sketching was calculated. The coefficient of determination (R^2^) used to compare frequencies prior to and after subsampling was calculated using the stat_poly_eq function from the ggpmisc library.

### Inference of Populations Using Logistic Regression

In order to transfer the subpopulations identified within the SP CD8ß^+^, logistic regression was performed using the LogisticRegression function from the sklearn python module in R using reticulate. The best parameters were assessed using the GridSearchCV function from the sklearn python module in R using reticulate. Following classification of the SP CD4^+^ using logistic regression, the accuracy defined as $\frac{\text{TP}+\text{TN}}{\text{TP}+\text{TN}+\text{FN}+\text{FP}}$, sensitivity $\frac{\text{TP}}{\text{TP}+\text{FN}}$ and specificity $\frac{\text{TN}}{\text{TN}+\text{FP}}$ were calculated.

### Statistical Analysis

Differences between discrete age groups were tested using linear regression where the day on which the samples were acquired on the flow cytometer was taken into account as a confounding variable. Differences between populations were tested using a paired *t*-test. The *p*-values were corrected for multiple testing using the p.adjust function.

### Data Visualization

All data was visualized using the ggplot2 library. Loess curves and boxplots were also generated using the ggplot2 library.

### Software

Data analysis was done using R (version 3.6.3) and python (version 3.7.3), while manual gating was done using FlowJo (version 10.0.7).

## Data Availability Statement

The original contributions presented in the study are included in the article/Supplementary Material, further inquiries can be directed to the corresponding author/s.

## Ethics Statement

Thymus from patients undergoing cardiac surgery were obtained and used according to and with the approval of the Medical Ethical Commission of Ghent University Hospital (Belgium).

## Author Contributions

BV performed experiments. ML performed bioinformatics analysis, designed the study, and wrote the paper. KL designed the study, wrote the paper, and supervised the study. TT designed the study, wrote the paper, supervised the study, and acquired funding. BV and GL provided expertise. All authors contributed to the article and approved the submitted version.

## Conflict of Interest

The authors declare that the research was conducted in the absence of any commercial or financial relationships that could be construed as a potential conflict of interest.
