# Supplementary material for: Conventional and Computational Flow Cytometry Analyses Reveal Sustained Human Intrathymic T Cell Development From Birth Until Puberty
Source: Front Immunol. 2020 Aug 4;11:1659. doi: 10.3389/fimmu.2020.01659 (PMC7417369; doi:10.3389/fimmu.2020.01659)
Supplement: Supplementary file 1 [file Data_Sheet_1.pdf]

Supplementary Material

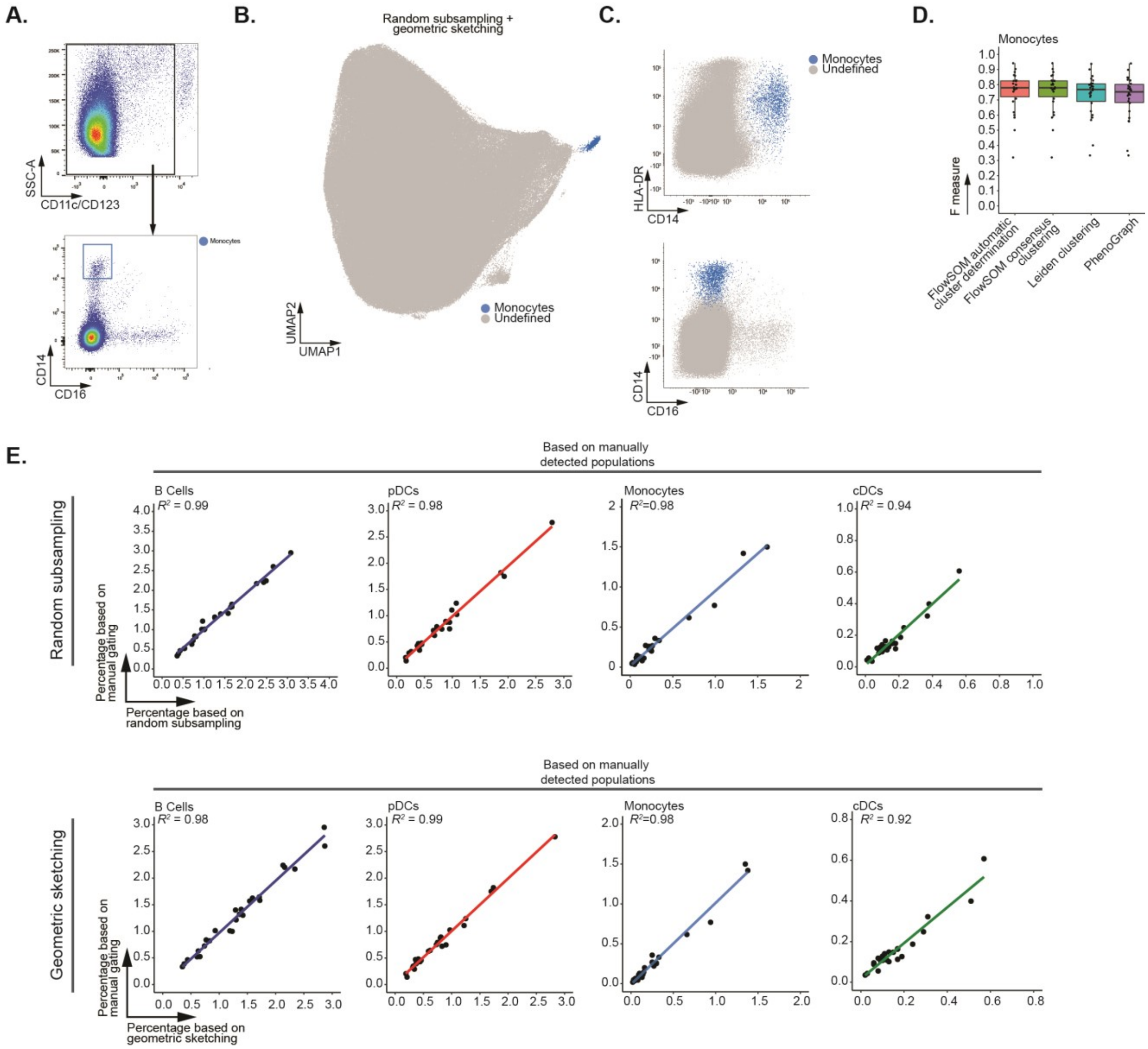

Figure S1 | Validation of graph-based clustering on subsampled data using myeloid thymocytes. **(A)** Identification of monocytes and macrophages using manually defined gates. **(B)** Annotated UMAP visualization derived from MNC samples (n = 26) following subsampling. **(C)** Scatterplots visualizing computationally identified monocytes and macrophages. **(D)** Boxplots visualizing F measures calculated for each MNC sample (n = 26) per analysis method. **(E)** Pairwise scatterplots visualizing frequencies of immune subsets identified manually and computationally (after random subsampling or geometric sketching). Accompanied by the coefficient of determination (R^2).

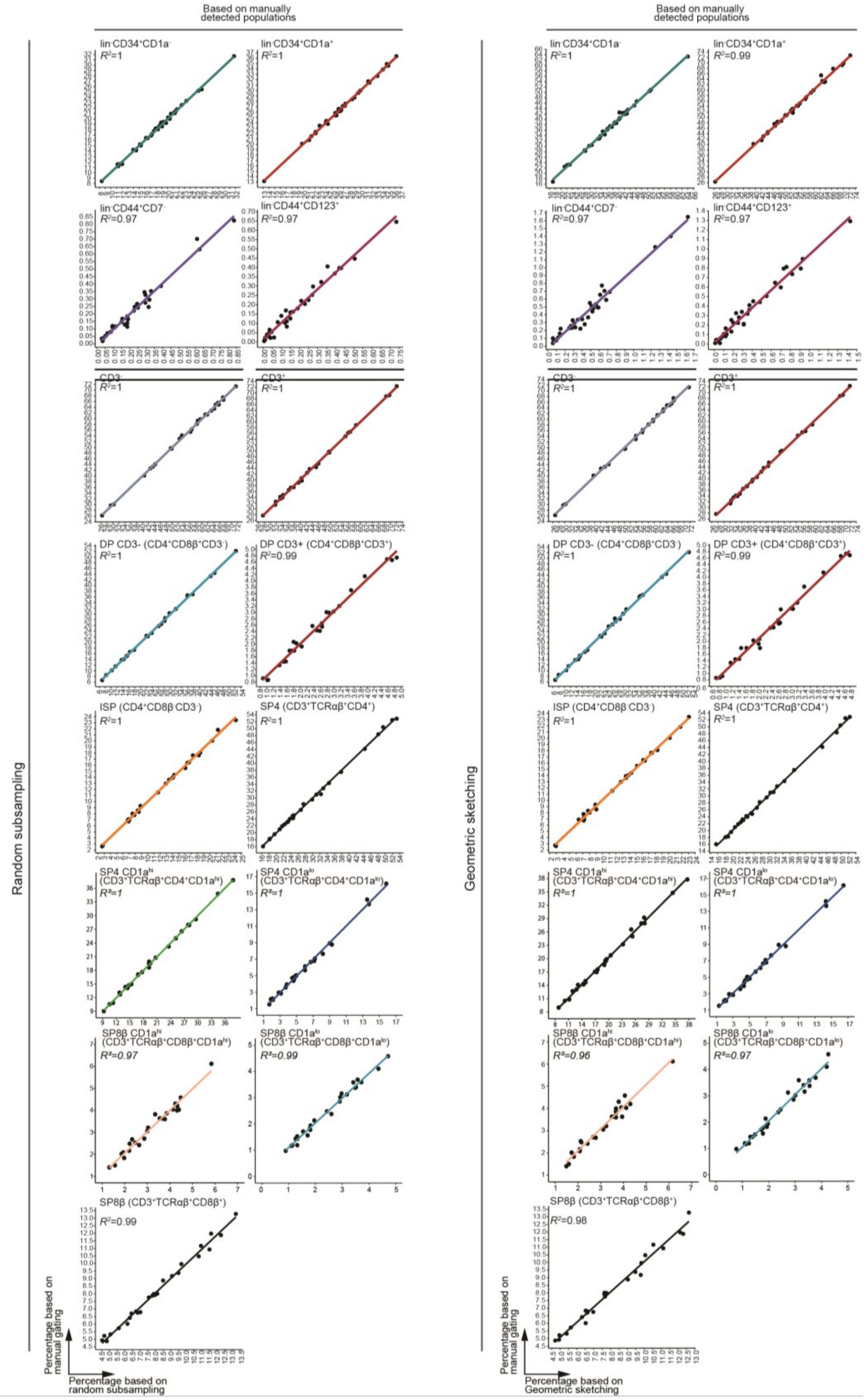

Figure S2 | Subsampling of the data does not affect calculation of population frequencies. Pairwise scatterplots visualizing frequencies of immune subsets manually identified prior to and after randomized subsampling. Accompanied by the coefficient of determination (R^2).

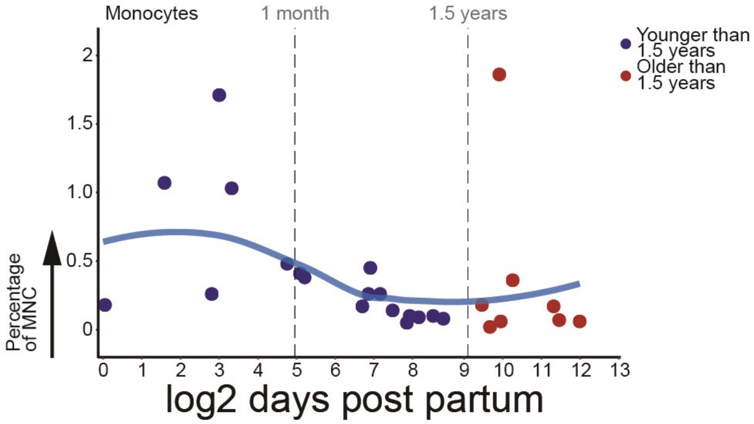

Figure S3 | Monocytes and macrophages population frequencies remain stable prior to puberty. Dot plot visualizing age-dependent changes in frequency with samples ordered along log2 transformed age within thymocyte populations. Color corresponds to patients younger (blue) or older than 1.5 years (red) within a population. A loess curve (span = 1) was fitted through the data in order to visualize the trend.

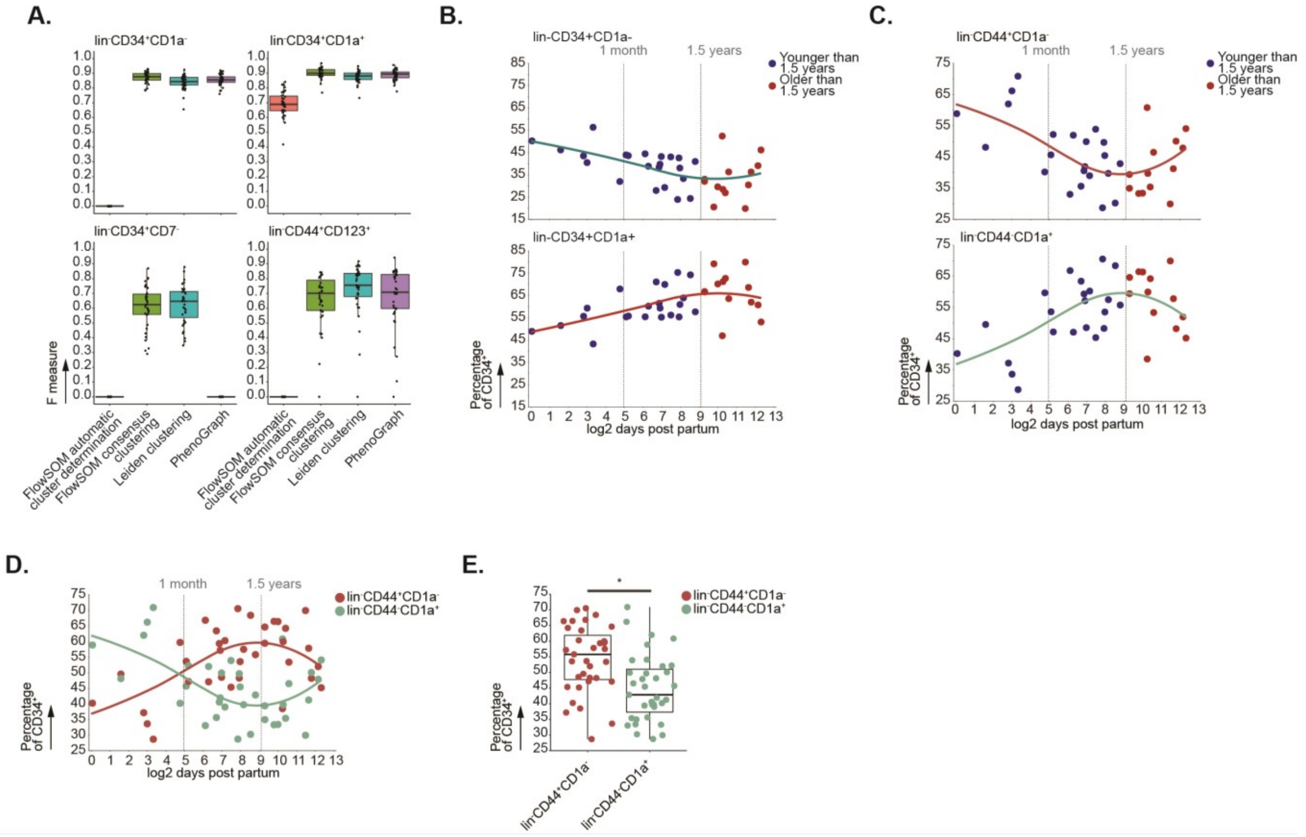

Figure S4 | The $CD34^{+}$ human thymocyte fraction is unaffected by aging up to puberty. **(A)** Boxplots visualizing F measures for each $CD34^{+}$ sample ($n = 35$) per analysis method and population. **(B,C)** Dot plots visualizing age-dependent changes in frequency with samples ordered along log2 transformed age within the $CD34^{+}$ thymocytes. Color corresponds to categories of patients younger (blue) or older than 1.5 years (red). A loess curve (span = 1) was fitted through the data in order to visualize the trend. **(D)** Dot plots visualizing age-dependent changes in frequency with samples ordered along log2 transformed age (days), comparing the $lin^{-}CD44^{+}CD1a^{-}$ and $lin^{-}CD44^{-}CD1a^{+}$ populations. A loess curve (span = 1) was fitted through the data in order to visualize the trend. **(E)** Boxplots visualizing discrete changes in frequency between the $lin^{-}CD44^{+}CD1a^{-}$ and $lin^{-}CD44^{-}CD1a^{+}$ populations. Statistical significance denoted as $*$ corresponds to a q-value <= 0.01.

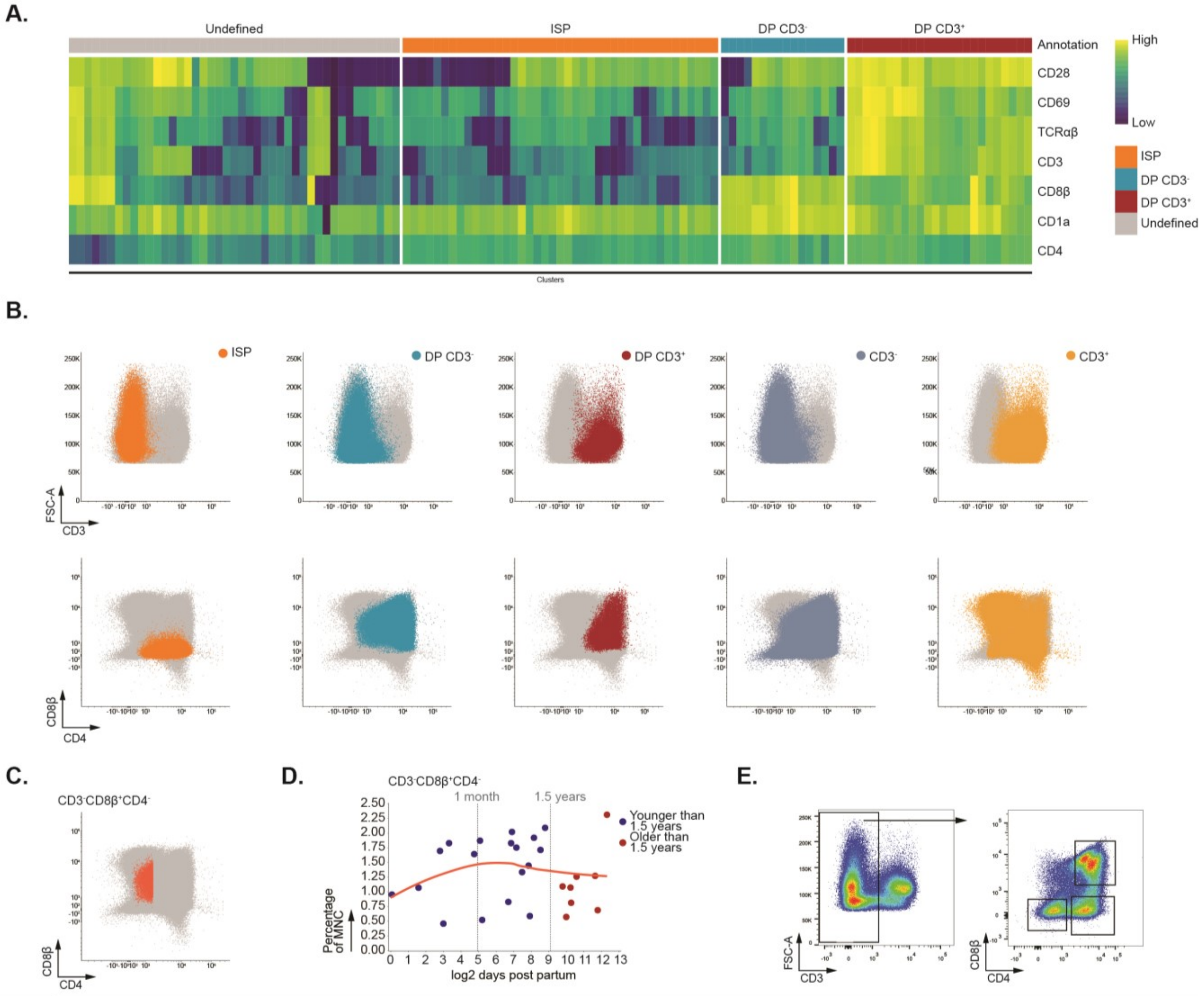

Figure S5 | The human thymus contains a population of ISP CD8β thymocytes **(A)** Heatmap visualizing the scaled MFIs for each cluster grouped according to their annotation. **(B)** Scatterplots of biexponentially transformed data visualizing the populations detected in **Figure 4B** using relevant markers. **(C)** Scatterplot of biexponentially transformed data visualizing a population of ISP CD8β$^+$ thymocytes. **(D)** Dot plot visualizing age-dependent changes in frequency with samples ordered along log2 transformed age within the MNC fraction. Color corresponds to categories of patients younger (blue) or older than 1.5 years (red). A loess curve (span = 1) was fitted through the data in order to visualize the trend. **(E)** Manual gating strategy used to identify T cell developmental intermediates prior to CD3 expression. Data visualized is biexponentially transformed.

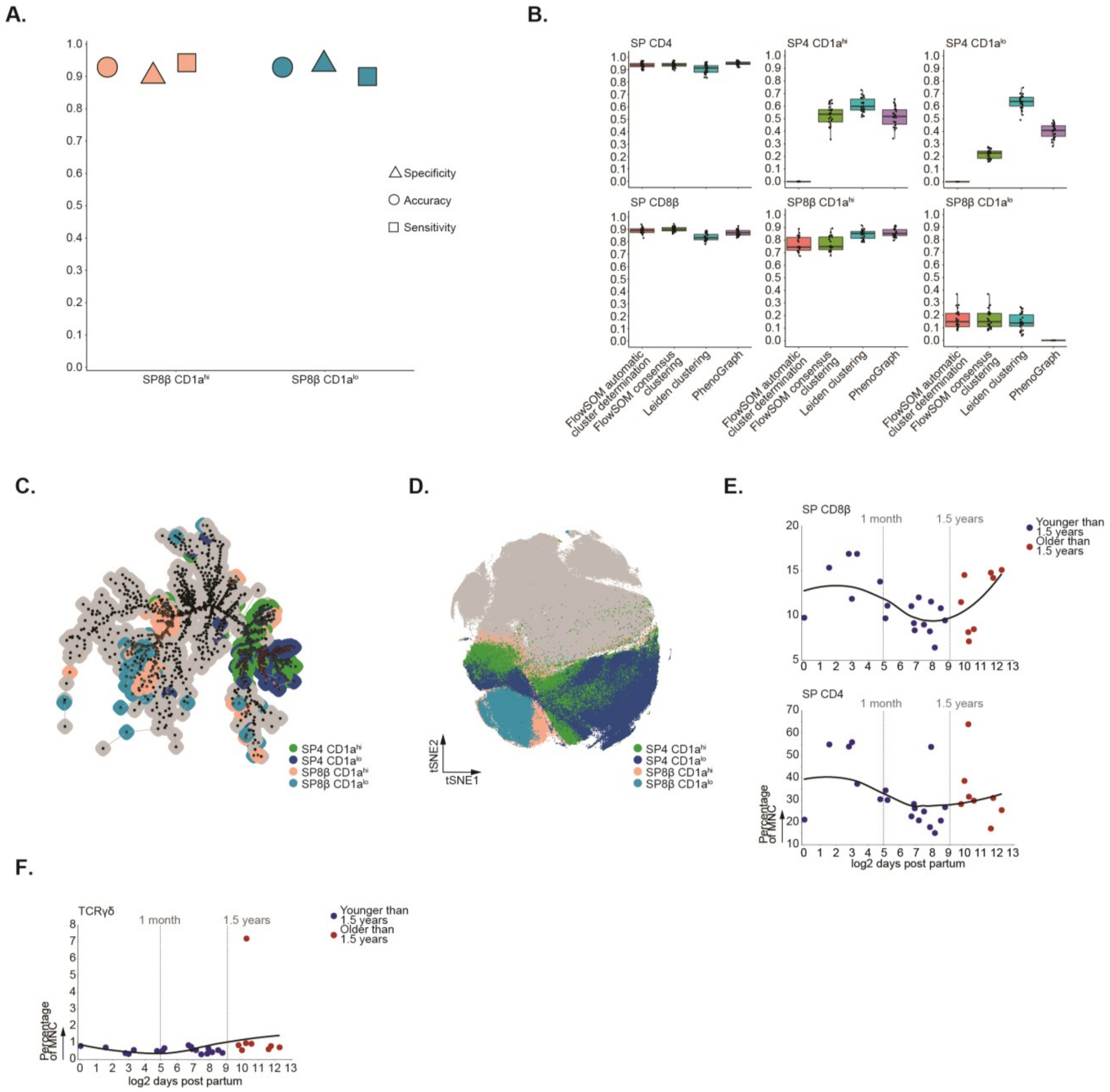

Figure S6 | Logistic regression allows for accurate inference of subpopulations. **(A)** Overview of the accuracy, specificity and sensitivity of the logistic regression model trained on the SP CD8β thymocytes. **(B)** Boxplots visualizing F measures for each of the MNC samples (n = 26) per analysis method and population. **(C,D)** Annotated MST **(C)** and tSNE visualizations **(D)** of the MNC thymocyte fraction visualizing mature αβ T cell populations as defined in **Figure 5A**. **(E,F)** Dot plots visualizing age-dependent changes in frequency with samples ordered along log2 transformed age within the MNC fraction. Color corresponds to categories of patients younger (blue) or older than 1.5 years (red). A loess curve (span = 1) was fitted through the data in order to visualize the trend.
